# Application of phase-contrast cine magnetic resonance imaging in endoscopic aqueductoplasty

**DOI:** 10.3892/etm.2013.1062

**Published:** 2013-04-10

**Authors:** GUOQIANG CHEN, JIAPING ZHENG, QING XIAO, YUNSHENG LIU

**Affiliations:** 1 Department of Neurosurgery, Xiangya Hospital, Central-South University, Changsha, Hunan 410008;; 2 Department of Neurosurgery, Yuquan Hospital, Tsinghua University, Beijing 100049, P.R. China

**Keywords:** aqueductoplasty, cerebrospinal fluid flow, dynamics, hydrocephalus, neuroendoscope, oculomotor paralysis, phase-contrast cine magnetic resonance imaging

## Abstract

The aim of this study was to evaluate the application of phase-contrast cine magnetic resonance imaging (MRI) in endoscopic aqueductoplasty (EA) for patients with obstructive hydrocephalus. The clinical diagnosis of hydrocephalus caused by aqueduct obstruction in 23 patients was confirmed by phase-contrast cine MRI examination. The patients were treated with EA and MRI was repeated during the follow-up. The cerebrospinal fluid (CSF) flow velocity in the aqueduct was measured to determine whether the aqueduct was obstructed. The results of phase-contrast cine MRI examinations indicated that there was no CSF flow in the aqueduct for all patients prior to surgery. Aqueductoplasty was successfully performed in all patients. The results of phase-contrast cine MRI examinations performed a week after surgery demonstrated an average CSF flow velocity of 4.74±1.77 cm/sec. During the follow-up, intracranial hypertension recurred in two patients in whom CSF flow was not observed in the aqueduct by the phase-contrast cine MRI scan. Aqueduct re-occlusion was revealed by an endoscopic exploration. By measuring the CSF flow velocity, phase-contrast cine MRI accurately identifies aqueduct obstruction. Cine MRI is a nontraumatic, simple and reliable method for determining whether the aqueduct is successfully opened following aqueductoplasty.

## Introduction

Endoscopic third ventriculostomy (ETV) is widely used to treat hydrocephalus caused by aqueduct obstruction (1–4). However, ETV has the risk of serious complications, including the rupture of the basilar artery and other injuries to the hypothalamus. Endoscopic aqueductoplasty (EA) has gradually become an alternative treatment for obstructive hydrocephalus (5,6). However, there are a limited number of studies on aqueductoplasty. There have been <50 reported cases since the 1990s. The average follow-up period is <3 years and the success rate ranges from 30 to 100% (5,7–11). EA may become an alternative option for patients with obstructive hydrocephalus due to aqueduct stenosis. However, an appropriate method must first be established to control the surgical indications prior to surgery and to evaluate the effectiveness of surgery. For two decades, phase-contrast cine magnetic resonance imaging (MRI) has been used to study the physiological state of the cerebrospinal fluid (CSF) circulation, to diagnose hydrocephalus and to evaluate the efficacy of third ventriculostomy. This type of MRI has been widely used by clinicians since it is non-invasive and highly sensitive (12–14). In this study, phase-contrast cine MRI was used for the preoperative diagnosis of obstructive hydrocephalus and the postoperative follow-up, with satisfactory outcomes.

## Patients and methods

### Patients

**Figure 1:**
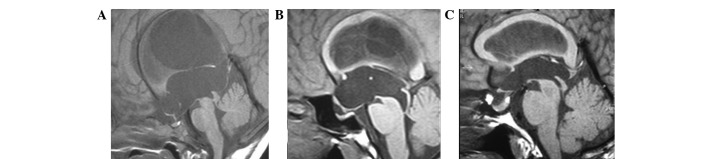
Mid-sagittal magnetic resonance imaging (MRI) revealed a membranous septum and the third ventricular floor was flat. (A and B) The aqueduct membranous obstruction revealed the goblet-like change. (C) The aqueduct membranous obstruction without the goblet-like change.

A total of 274 patients with obstructive hydrocephalus who underwent endoscopic neurosurgery between February 2007 and August 2009 were included in our study. Among these patients, 23 had aqueduct reconstruction (Table I). The following criteria were used to select patients for aqueductoplasty (Fig. 1): i) all patients with membranous obstruction of the aqueduct and ii) all cases with thickening of the ventricle floor.

Twenty-one cases had simple membranous obstruction at the aqueduct fistula, whereas two cases had hydrocephalus due to intraventricular hemorrhage. In total, 10 males and 13 females underwent aqueductoplasty, with an average age of 10.5 years (the patients’ age ranged from 3 months to 67 years). Prior to surgery, the patients had a variety of clinical symptoms, which included headaches, nausea, vomiting, blurred vision, unstable movement, an increased head circumference and a loss of consciousness. A preoperative phase-contrast cine MRI scan confirmed the aqueduct obstruction and the cessation of CSF flow in the aqueduct. This study was conducted in accordance with the Declaration of Helsinki. This study was conducted with approval from the Ethics Committee of Xiangya Hospital, Central-South University (Changsha, China). Written informed consent was obtained from all participants.

### Phase-contrast cine MRI scans

**Figure 2:**
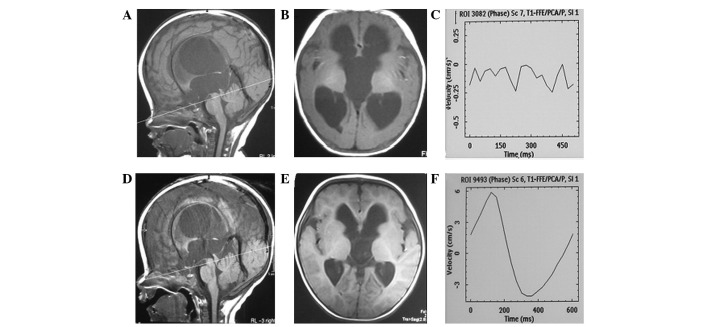
(A) Sagittal and (B) coronal magnetic resonance imaging (MRI) prior to neuroendoscopic aqueductoplasty (EAP); (C) Cerebrospinal fluid (CSF) flow velocity at the aqueduct fistula level measured by phase-contrast MRI prior to EAP. (D) The postoperative image demonstrates a patent aqueduct with a flow void. (E) Scan following EAP revealed reduced ventricular size, cephalus and membranous obstruction with prestenotic dilatation. (F) CSF flow velocity at the aqueduct fistula level measured by phase-contrast MRI following aqueductoplasty. Quantification of CSF flow was performed in the transverse plane perpendicular to the midcollicular level.

All subjects underwent conventional head MRI examination with a 1.5 Tesla MRI scanner (Signa Horizon MRI system; GE Healthcare, Piscataway, NJ, USA). The scanning sequence included the T1WI, T2WI and fluid attenuated inversion recovery (FLAIR) sequences. The following scan parameters were set for the axial position: repetition time/echo time (TR/TE), 450/12 msec; field of view (FOV), 27×27 cm^2^; slice thickness, 8 mm; interval, 0.8 mm; and matrix, 256×256; whereas for the sagittal position, the parameters were: TR/TE, 360/12 msec; FOV, 30×30 cm^2^; slice thickness, 5 mm; interval, 0.6 mm; and matrix, 256×192. The sagittal scout view sequences were used as localizers to select the anatomic levels for the flow velocity measurements. The CSF flow velocity was measured in the transverse plane perpendicular to the mid-collicular level of the aqueduct (Fig. 2), in terms of the peak systolic or diastolic velocity. The aqueduct was identified and the circular region of interest (ROI) was placed inside it. For the GE MRI phase-contrast cine analysis system, the following scan parameters were used in the axial position: TR/TE at the minimum; FOV, 28×28 cm^2^; slice thickness, 10 mm; matrix, 256×192; number of excitations (NEX), 2; flip angle, 15°; velocity encoding (Venc), 10 cm/sec; and slice number, 9; whereas the parameters in the sagittal position were: TR/TE at the minimum; FOV, 14×14 cm^2^; section thickness, 5 mm; matrix, 256×192; NEX, 2; flip angle, 15°; Venc, 10 cm/sec; and slice number, 20. The encoding direction was from the top to the bottom. The peripheral gating was selected for the scans without slice-overlap, respiratory compensation or flow compensation. The total scanning time was 20–25 min.

### Surgical equipment

Surgical equipment included a 3.8 mm Rudolf-Fujinon flexible electronic endoscope (Fujifilm Corporation, Tokyo, Japan), a set of matched single and bipolar coagulation devices, pairs of biopsy forceps and scissors and a 2F Fogarty balloon catheter.

### Surgical procedure

**Figure 3:**
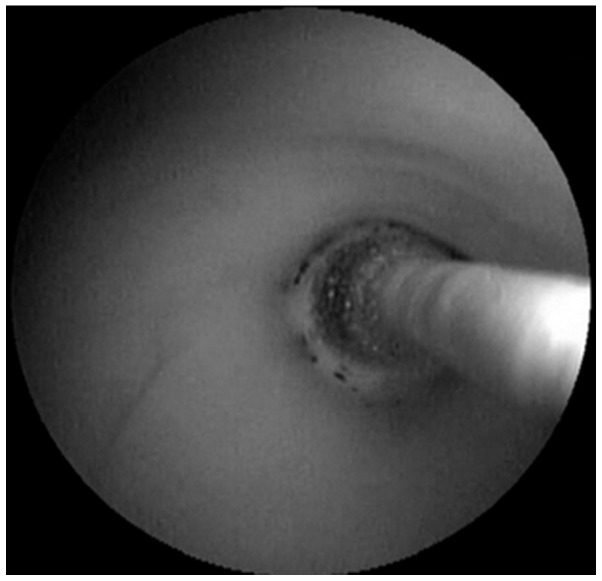
Dilatation of the stenosis with a Fogarty balloon catheter.

Under general anaesthesia, patients were placed in the supine position with the head tilted at 30°. A scalp incision was made above the forehead hairline and 2 cm from the two sides of the median line. A 2-cm hole was drilled into the skull and a sheath was used to puncture the lateral ventricle. A ventriculoscope was inserted into the ventricle to view the aqueduct fistula. The catheter was pushed through the membranous obstruction and short aqueductal stenosis such that its balloon portion was at the lower fistula of the aqueduct. The balloon was filled with 0.1–0.2 ml normal saline to expand the aqueduct to a diameter of 4 mm (Fig. 3). Then, the endoscope was inserted further into the fourth ventricle to explore whether the foramen of Luschka was obstructed (15,16).

### Follow-up

All patients underwent a routine phase-contrast cine MRI scan one week after surgery to determine whether obstruction of the aqueduct remained. The mean follow-up duration was 19 months (range, 16–32 months). All patients returned for follow-up after >1 year and the phase-contrast cine MRI was repeated. Two patients immediately had MRI scans when the symptoms of intracranial hypertension recurred.

### Statistical analysis

Statistical evaluation of the data was performed using a commercially available software package (SPSS System for Windows, version 17.0; SPSS Inc., Chicago, IL, USA). The mean and standard deviation were calculated for each parameter. The t-test was used for comparisons of different age groups. P<0.05 was considered to indicate a statistically significant difference.

## Results

**Figure 4:**
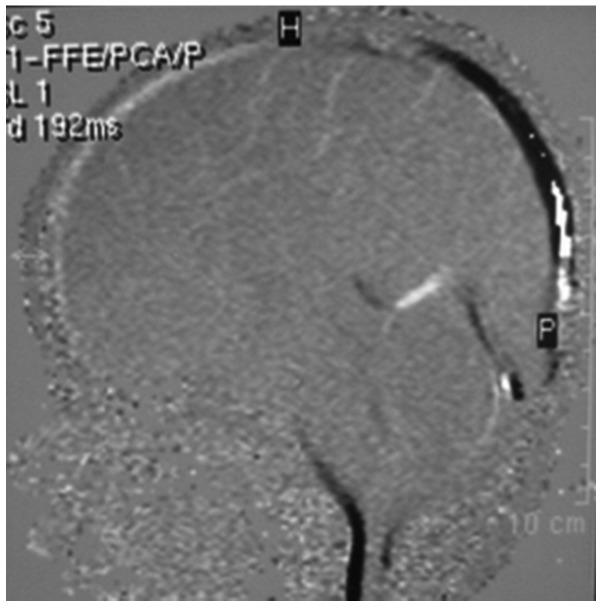
Midsagittal phase-contrast image. There was no cerebrospinal fluid through the aqueduct.

Complete aqueduct obstruction was revealed by preoperative phase-contrast cine MRI in 23 patients (Figs. 2 and 4). During surgery, a membranous obstruction at the upper aqueduct, as well as short aqueductal stenosis, were observed in 21 patients. In two patients with intraventricular hemorrhage, the opening of the upper aqueduct was occluded by old blood clots. Approximately one week after surgery, smooth CSF flow was observed by phase-contrast cine MRI at the aqueduct opening. In the postoperative MRI scans of the CSF flow, the flow-void signal phenomenon was observed in 14 patients (Fig. 4). The average peak flow velocity was 4.74±1.77 cm/sec. The typical flow velocity waveform for each cardiac cycle was a two-way flow (Fig. 2). In 21 patients, the simple membranous obstruction did not recur during follow-up. The ventricular size was reduced in eight patients, whereas no changes were observed in the other patients. The one-year follow-up MRI scans revealed that the CSF flow was smooth in all patients. The average peak flow velocity was 4.28±2.17 cm/sec. The flow velocity waveform for each cardiac cycle was bi-directional.

Symptoms recurred during follow-up in the two patients with hydrocephalus due to intraventricular hemorrhage. One patient had symptoms of intracranial hypertension one month after aqueductoplasty. The MRI scans revealed that there was no CSF flow in the aqueduct and a second endoscopic examination revealed that the aqueduct opening was covered by old blood clots and a proliferative ventricular membrane. After the obstruction was relieved, the aqueduct was observed to be recanalized on repeated phase-contrast cine MRI. In the second patient, the symptoms of intracranial hypertension recurred three months after aqueductoplasty. There was no CSF flow in the aqueduct, as revealed by the phase-contrast cine MRI. A second endoscopy examination confirmed restenosis in the aqueduct. The aqueduct was expanded and then a stent was placed. The patient was free of symptoms following stenting.

Of the 23 patients, only one patient reported oculomotor nerve palsy. This patient recovered after three months.

The average peak flow velocity one week after surgery was similar for patients aged <2 years (4.96±1.83 cm/sec) and those who were older than 2 years (4.53±1.75 cm/sec) (P>0.05). The average peak flow velocity, as measured at one year after surgery, remained similar in patients aged <2 years (4.60±2.26 cm/sec) and those older than 2 years (3.97±2.13 cm/sec; P>0.05).

## Discussion

In 1920, Dandy (15) used a probe to perform aqueduct reconstruction through the fourth ventricle for the first time. With the development of endoscopic equipment and technology, third ventriculostomy has been widely used to treat patients with hydrocephalus due to aqueduct stenosis. The procedure is relatively safe and has fewer complications than other methods. In the majority of cases, this procedure helps to wean patients off shunt devices. However, certain fatal complications, including injuries to the basilar artery, have been reported for third ventriculostomy (17). In addition, a thick or tough bottom in the third ventricle makes ventriculostomy difficult to perform. Moreover, stenosis at the bottom of the third ventricle may damage the hypothalamus or pituitary stalk. Therefore, EA is used as an alternative option for treating patients with membranous obstruction. When the preoperative MRI sagittal view reveals that the third ventricle floor is flat or when the coronal MRI view reveals that the third ventricular is narrow, the floor of the third ventricle may be difficult to penetrate during surgery (Fig. 1). However, judgments that are based on the findings of MRI imaging are not always reliable. The preoperative MRI revealed that 21 patients had a membranous obstruction of the proximal aqueduct outlet (Fig. 1). The thickness of the membrane was <1 mm, as measured by MRI. Two patients had a short obstruction that was <3 mm. According to the results of the preoperative mid-sagittal MRI, 21 cases had occlusions in the proximal inlet of the aqueduct, in which the occluding membrane was <1 mm (Fig. 1). Two cases had short segmental stenosis of ∼3 mm.

Phase-contrast cine MRI provides important information concerning the hemodynamics of the CSF circulation. The earliest MRI examinations only provide a qualitative description of CSF flow in the stenotic aqueduct or ventricles through the CSF flow-void signs. Magnetic resonance T1- or T2-weighted images improve a visualization of the anatomical structure prior to surgery. In our study, phase-contrast MRI was used to screen patients prior to surgery. Our results indicated that the diagnosis of aqueduct obstruction was consistent with the intra-operative exploration in all cases.

**Figure 5:**
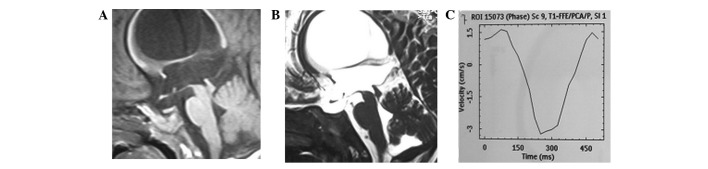
Magnetic resonance imaging (MRI) of a patient one year after surgery shown in: (A) sagittal T1-weighted MR image; the cerebrospinal fluid (CSF) flow-void sign within the aqueduct and fourth ventricle were not observed; (B) sagittal T2-weighted MR image revealed re-occlusion of the aqueduct. (C) CSF flow waveform showing a flow pattern revealed that the aqueduct was open.

No specific standards exist for the evaluation of EA efficacy. To date, the only standard that is recognised by the majority of researchers is shunt independence. Moreover, short-term follow-up does not identify delayed surgical failure (8). In certain patients, particularly in pediatric patients with chronic obstructive hydrocephalus, the clinical symptoms are not evident within a short period of time. By contrast, long-term observation may delay the best time for treatment. The findings from MRI are necessary to investigate whether the aqueduct is successfully opened following surgery (6,13,18). In the majority of cases, a sufficient CSF flow is observed as a flow-void signal in individual cases with relieved symptoms. However, from the sagittal T1- and T2-weighted images, the CSF flow-void signs within the aqueduct and the fourth ventricle are not observed. The CSF flow waveform shows the respective flow pattern, which indicates that the aqueduct is open (Fig. 5). In the majority of cases in this study, the flow-void signals of CSF were detected following surgery. In a few cases with clinical improvement following surgery, the flow void was not detected; however, the CSF cine images revealed that the aqueduct was opened following surgery.

The imaging evaluation criteria generally include the reduction of the ventricle size and subarachnoid space, the absorption of periventricular edema and the CSF flow-void signal phenomenon. However, 50% of patients with improved symptoms have little or no significant changes in ventricle size following surgical treatment. The absorption of periventricular edema remains controversial (9). The subarachnoid width may only be used as a secondary indicator. In addition, the CSF flow-void signal phenomenon is not observed in a number of patients. Previous reports indicated that ventricle size did not change in 11–38% of patients who received third ventriculostomy (13,14).

Schroeder *et al*(6) assessed the CSF circulation by phase-contrast MRI in 14 healthy volunteers and eight patients who underwent EA. No significant differences in the CSF time index, peak velocity, average flow velocity or volume between the two groups were observed, which suggested that the CSF circulation was normal following aqueductoplasty.

EA is an important alternative treatment for obstructive hydrocephalus when third ventriculostomy is difficult to perform. However, the success rate varies in different reports due to the small sample sizes and the short follow-up times. The intraoperative implantation of a stent remains controversial. Thus, the evaluation of postoperative efficacy becomes particularly important. In addition to symptom relief, imaging results are essential for the evaluation of postoperative efficacy. Phase-contrast MRI has been used to study CSF circulation for >20 years. This technique is widely used for the clinical diagnosis of hydrocephalus. Kim *et al*(19) recommended phase-contrast MRI as a significant method for the evaluation of the position and severity of obstruction in patients with obstructive hydrocephalus. In the current study, the results of post-aqueductoplasty phase-contrast MRI indicated that the aqueduct was unobstructed in 23 patients. During follow-up, the reduction in ventricle size was observed in eight patients and the CSF flow-void signal phenomenon was observed in four patients. In two patients with recurrence of intracranial hypertension, phase-contrast MRI did not show any CSF flow inside the aqueduct. The second endoscopic exploration revealed that the aqueduct was closed. The test results were consistent with the clinical findings.

**Figure 6:**
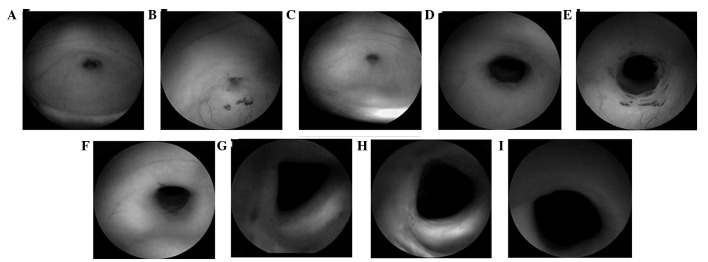
(A,B,C) Membrane occluding the proximal inlet of the aqueduct; the membranous obstruction is translucent. (D,E,F) The stoma to the fourth ventricle following neuroendoscopic aqueductoplasty (EAP). (G,H,I) Intracanalicular structures with no injury. (A,D,G) were from one patient, (B,E,H) were from another patient and (C,F,I) were from another patient.

Enchev *et al*(20) proposed that EA carries potential risks that should not be underestimated. The authors demonstrated that direct surgery-related complications, including damage to the fornix, aqueductal roof or floor of the third ventricle, venous-arterial bleeding and particularly injury to the eloquent periaqueductal grey structures may occur; these complications should be carefully considered prior to surgery. In 21 patients, the obstructive hydrocephalus was caused by the membrane that occluded the proximal inlet of the aqueduct. The fiber-scope should be set at a right angle to the inlet of the aqueduct. Fenestration of the septum was carefully performed using the balloon technique. Opening a thin membrane is a relatively easy and safe neuroendoscopic procedure (Fig. 6). Compared with a membranous septum, short segmental stenosis is more difficult to conduct surgery on. The potential risk of injury to the midbrain is associated with the length of the stenosis (10).

ETV has become the first line treatment option for obstructive hydrocephalus; however, aqueductoplasty may be used when surgery using ETV would be difficult. In certain cases, EA should be considered as an alternative treatment option in patients with short or membranous stenosis of the rostral aqueduct. The potential risk of injury to the midbrain and periaqueductal grey matter is low. Phase-contrast cine MRI may be a valuable tool in the evaluation of a hydrocephalic ventricle system.

## Figures and Tables

**Table I:** Summary of 23 patients.

Patient	Gender/age (months or years)	Diagnosis	Symptoms	Surgical outcome
1	F/9 y	AO	Headache, vomiting	Improved
2	M/9 m	AO	Increased head circumference	Improved
3	F/17 m	AO	Increased head circumference	Improved
4	M/5 y	AO	Headache, vomiting	Improved
5	M/5 y	AO	Increased head circumference	Improved
6	F/5 y	AO	Increased head circumference	Improved
7	F/2 y	AO	Increased head circumference	Improved
8	M/18 m	AO	Increased head circumference	Improved
9	M/19 m	AO	Increased head circumference	Improved
10	F/33 y	AO	Headache, vomiting	Improved
11	M/9 m	IVH	Instability of movement	Failure
12	F/3 y	AO	Instability of movement	Improved
13	F/16 m	AO	Increased head circumference	Improved
14	M/26 y	AO	Headache, vomiting	Improved
15	F/28 y	AO	Headache, vomiting	Improved
16	F/3 m	IVH	Increased head circumference	Failure
17	M/11 m	AO	Increased head circumference	Improved
18	M/20 y	AO	Headache, vomiting	Improved
19	F/24 y	AO	Headache, vomiting	Improved
20	F/67 y	AO	Blurred vision, loss of consciousness	Improved
21	F/6 m	AO	Increased head circumference	Improved
22	F/16 m	AO	Increased head circumference	Improved
23	M/17 m	AO	Increased head circumference	Improved

AO, aqueductal obstruction; IVH, intraventricular hemorrhage.

## References

[1] Jenkinson MD, Hayhurst C, Al-Jumaily M, Kandasamy J, Clark S, Mallucci CL (2009). The role of endoscopic third ventriculostomy in adult patients with hydrocephalus. J Neurosurg.

[2] Kulkarni AV, Drake JM, Mallucci CL (2009). Endoscopic third ventriculostomy in the treatment of childhood hydrocephalus. J Pediatr.

[3] Ogiwara H, Dipatri AJ, Alden TD, Bowman RM, Tomita T (2010). Endoscopic third ventriculostomy for obstructive hydrocephalus in children younger than 6 months of age. Childs Nerv Syst.

[4] Sacko O, Boetto S, Lauwers-Cances V, Dupuy M, Roux FE (2010). Endoscopic third ventriculostomy: outcome analysis in 368 procedures. J Neurosurg Pediatr.

[5] Ersahin Y (2007). Endoscopic aqueductoplasty. Childs Nerv Syst.

[6] Schroeder HW, Schweim C, Schweim KH, Gaab MR (2000). Analysis of aqueductal cerebrospinal fluid flow after endoscopic aqueductoplasty by using cine phase-contrast magnetic resonance imaging. J Neurosurg.

[7] da Silva LR, Cavalheiro S, Zymberg ST (2007). Endoscopic aqueductoplasty in the treatment of aqueductal stenosis. Childs Nerv Syst.

[8] Fritsch MJ, Kienke S, Mehdorn HM (2004). Endoscopic aqueductoplasty: stent or not to stent?. Childs Nerv Syst.

[9] Garg AK, Suri A, Sharma BS, Shamim SA, Bal CS (2009). Changes in cerebral perfusion hormone profile and cerebrospinal fluid flow across the third ventriculostomy after endoscopic third ventriculostomy in patients with aqueductal stenosis: a prospective study. Clinical article. J Neurosurg Pediatr.

[10] Miki T, Nakajima N, Wada J, Haraoka J (2005). Indications for neuroendoscopic aqueductoplasty without stenting for obstructive hydrocephalus due to aqueductal stenosis. Minim Invasive Neurosurg.

[11] Oertel JM, Baldauf J, Schroeder HW, Gaab MR (2009). Endoscopic options in children: experience with 134 procedures. J Neurosurg Pediatr.

[12] Alperin N, Vikingstad EM, Gomez-Anson B, Levin DN (1996). Hemodynamically independent analysis of cerebrospinal fluid and brain motion observed with dynamic phase contrast MRI. Magn Reson Med.

[13] Bargalló N, Olondo L, Garcia AI, Capurro S, Caral L, Rumia J (2005). Functional analysis of third ventriculostomy patency by quantification of CSF stroke volume by using cine phase-contrast MR imaging. AJNR Am J Neuroradiol.

[14] Stivaros SM, Sinclair D, Bromiley PA, Kim J, Thorne J, Jackson A (2009). Endoscopic third ventriculostomy: predicting outcome with phase-contrast MR imaging. Radiology.

[15] Dandy WE (1920). The diagnosis and treatment of hydrocephalus resulting from strictures of the aqueduct of Sylvius. Surg Gynecol Obstet.

[16] Schroeder HW, Gaab MR (1999). Endoscopic aqueductoplasty: technique and results. Neurosurgery.

[17] Lipina R, Reguli S, Dolezilová V, Kuncíková M, Podesvová H (2008). Endoscopic third ventriculostomy for obstructive hydrocephalus in children younger than 6 months of age: is it a first-choice method?. Childs Nerv Syst.

[18] Schroeder HW, Oertel J, Gaab MR (2007). Endoscopic treatment of cerebrospinal fluid pathway obstructions. Neurosurgery.

[19] Kim MH, Shin KM, Song JH (1998). Cine MR CSF flow study in hydrocephalus: what are the valuable parameters?. Acta Neurochir Suppl.

[20] Enchev Y, Oi S (2008). Historical trends of neuroendoscopic surgical techniques in the treatment of hydrocephalus. Neurosurg Rev.

